# INTEREST GROUP SESSION—LIFELONG DISABILITIES: DEFINING HEALTH OUTCOMES FOR ADULTS AGING WITH DISABILITY

**DOI:** 10.1093/geroni/igz038.1275

**Published:** 2019-11-08

**Authors:** Kelly Munly, Caitlin E Coyle, Caitlin E Coyle

**Affiliations:** 1 Penn State Altoona, Altoona, Pennsylvania, United States; 2 Gerontology Institute, University of Massachusetts Boston, Boston, Massachusetts, United States

## Abstract

The population of persons aging with disabilities is growing. Being able to segment aging with disability sub-populations within national data sets is becoming increasingly important in order to understand the relationship of aging with disability to a range of outcomes in later life including health and wellness, economic security, and health and long-term service and support need and use. This symposium includes four examples of how existing data can be used to draw conclusions about the experience of old age for persons with intellectual or developmental disabilities. In addition, the symposium offers insights into the limitations of these data and the presentations lend themselves to a discussion of how measurement across disability sub-population can be ope rationalized. Two of the presentations focus on understanding mortality trends of adults with cerebral palsy and down syndrome--including an understanding of the health conditions facing these populations. A third presentation will focus on cardiovascular risk factors and co morbidity among adults with cerebral palsy. Finally, a fourth presentation will focus on pairing qualitative understanding with quantitative trends to offer a deeper understanding of the health management challenges for adults with disability as they age. Through a deeper understanding of the experience of health in later life for adults with disability, ideas about interventions and supports can be better aligned with the needs of these populations.

